# Learner-content interaction in e-learning- the moderating role of perceived harm of COVID-19 in assessing the satisfaction of learners

**DOI:** 10.1186/s40561-021-00149-8

**Published:** 2021-04-12

**Authors:** Pardeep Kumar, Charu Saxena, Hasnan Baber

**Affiliations:** 1 University School of Business, Chandigarh University, Mohali, India; 2 Endicott College of International Studies, Woosong University, Daejeon, South Korea

**Keywords:** E-learning, Quality, Learning content, Learners’ satisfaction, Website content, COVID-19

## Abstract

Envisioning learning sans interaction is absurd. Interaction plays a pivotal role in the efficacy and effectiveness of the present-day blended learning systems. Learner-content interaction contributes predominately towards the successful realization of the expected learning outcomes. In order to satisfy the learners and to impart them quality knowledge and education, e-learning content comprising of excellent learning and website content is of paramount importance. In the present COVID-19 outbreak challenging times the significance of e-learning system development and its application is much more pronounced. To gauge this, the study aims to examine the relationship between learner-content and the e-learning quality to determine the impact of e-learning quality on learners’ satisfaction under the moderating effect of perceived harm due to COVID-19. A structured questionnaire was used to gather data from 435 graduate and undergraduate management students (International and national) in Indian Universities. Findings indicate statistically significant relationships between the e-learning content and e-learning quality and; e-learning quality and the students’ satisfaction. The perceived harm has an insignificant moderating effect on students’ satisfaction. The results of the study further depict that the quality of e-learning has a significant positive relationship with the students’ satisfaction, and this relationship is not affected by the threat of being infected on the campus during the pandemic of COVID-19. To achieve the learners’ satisfaction, the institutions should strive for rendering the e-learning content of supreme quality. The mediating role of e-learning quality between content and students’ satisfaction is also established to be a significant one.

## Introduction

The twenty-first century witnessed an educational paradigm shift, across higher education institutions, with the advancement of information technologies. With continued improvement in the quality and scope of the e-learning content delivery, it is widely accepted that online networks are used as learning platforms that are widely spread, more flexible, easily accessible, and, most importantly, perpetually open. In the current period of the pandemic of COVID-19 breakdown, the blended learning system is a new and emerging, rapidly evolving way of teaching adopted by the various colleges and universities. Most higher education institutions have adopted the new learning system to guarantee less disruption of teaching and learning activities (Chin et al., 2020), even in this adverse pandemic period. The new learning process eliminates the barriers of distance and has shown an exponential enrollment of students in the various institutions, contributing towards the formation of a knowledgeable society (Taylor, 2007). On the other side, the task is equally challenging for the instructors to move from the physical classroom teaching to virtual classrooms towards ensuring effective interaction with the students (Kebritchi, Lipschuetz, & Santiague, 2017).

Student interaction is an important factor of any mode of learning, especially in the e-learning experience (Bernard et al., 2009), and the online interaction can occur in various modes like interaction with the instructor, interaction with peers, or interaction with the course content (Moore, 1993). Ultimately, the goal is qualitative learning and satisfaction of the learners. E-content can be effective if it can increase the learners’ understanding (Thurmond & Wambach, 2004) and able to change the students’ perspective. E-content is a package of course readings, multimedia links for demonstration, simulations, elaborative explanations; case studies, course assignments, discussion forums, having the potential to promote learning. As listening and reading, only, cannot affect cognitive learning and generate knowledge, (Bednar, Cunningham, Duffy, & Perry, 1992); it is possible through more and more interaction of students with the content which is designed to engage students in the online learning environment. Learner-content interaction typically occurs when, after listening to a demonstration on a particular topic, students go through the course readings, attempt the assignments given and participate in the online discussions; following the process of expressing, pondering, and exchanging their indulgences of course content (Jonassen, Davidson, Collins, Campbell, & Haag, 1995).

Due to the current pandemic period of COVID-19, in order to minimize the spread of the deadly virus, the majority of colleges and universities have opted to start the curriculum through an online learning system. Hodges, Moore, Lockee, Trust, and Aaron Bond (2020) called this shift towards e-learning during the pandemic and lockdown an *emergency remote teaching* (ERT). Thus, there is a swelling desire to understand the best way to engage the learners with both course content and their peers. Huang et al. (2020) suggested that there were three challenges faced by the instructors in e-learning during this pandemic- Lack of preparation time, Teacher/student isolation (first time for classroom learning) and the need for effective pedagogical approaches. Truhlar, Williams, and Walter (2018) examined a case study to assess the impact of assigning chat roles, group discussions on student-content engagement. The different tasks of assigning roles and discussion forums result in a high proportion of student-students interactions as well as student-content interactions. Moreover, rotation of role plays in the discussion forums and subsequent role assignment improves the students’ listening behavior (Wise & Chiu, 2014).

Learners’ satisfaction reflects how they view their learning experience, which is one of the crucial elements to assess the effectiveness of e-learning quality (Alqurashi, 2018). Although many researchers have assessed the satisfaction level of students with the online courses and e-learning environment, due to the overall dependence of the students on the e-learning system when the whole world is facing the disaster of COVID-19, it has become more important to understand the impact of e-learning quality on learners’ satisfaction. It is significant for the content designers and the instructors to know the perceived level of satisfaction of the learners with the current content and e-learning quality provided under the online study environment. In addition, as the perceived harm due to the outbreak of Coronavirus has enforced the students to embrace online learning, so it has become interestingly important to examine the moderating effect of perceived harm of getting COVID-19 on the relationship between e-learning quality and learners’ satisfaction.

In this paper, we aim to examine the relationships among e-learning content, overall e-learning quality, and learners’ satisfaction, along with the moderating effect of the perceived harm of getting COVID-19 on the satisfaction level of learners, in the context of the online learning system in the universities. More specifically, the current study aims to (1) assess the impact of e-learning content on learners’ satisfaction; (2) investigate the relationship between e-learning content and overall e-learning quality; (3) explore the relationship between overall e-learning quality and learners’ satisfaction; (4) investigate the moderating effect of perceived harm of getting COVID-19 on the relationship between e-learning quality and learners’ satisfaction, and (5) analyze the mediating role of E-learning Quality between Content and Learners’ Satisfaction.

## Theoretical and conceptual framework

Learning may be defined as the complex and long-term psychosocial process consisting of the individual acquisition or modification of information, knowledge, understanding, attitudes, values, skills, competencies or behaviours through experience, practice, study or instruction (UIS, 2012). E-learning can be defined as education based on modern methods of communication including the computer and its networks, various audio-visual materials, search engines, electronic libraries, and websites, delivered through the medium of the World Wide Web (Sotiriou, Lazoudis, & Bogner, 2020). It is a platform where the educational institution makes its programs and materials available on a special website in such a manner that students are able to make use of them and interact with them with ease through closed or shared, networks, or the Internet, and through use of e-mail and online discussion groups. This following segment encompasses the relevant literature on understudy variables along with their interdependence, as given under:

### E-learning content (*eLC*) and e-learning satisfaction (*SAT*) of the learners

Interaction acknowledged as an essential factor towards e-learning goals realization, has three vital dimensions, including learner to course-content, learner to the instructor, and learner to learner (Moore, 1993). Later, learner-interface interaction has also been added to these. Learning content is broadly defined as the topics, themes, beliefs, behaviors, concepts and facts, often grouped within each subject or learning area under knowledge, skills, values and attitudes, that are expected to be learned and form the basis of teaching and learning (UNESCO-IBE, 2013). Further, by e-learning content, we mean any document file, presentation file, audio file or video file that may be used to deliver e-learning. This includes power-point presentations, guides, reports, whitepapers, charts and graphs, illustrations, videos, case studies, infographics, problem-solution scenarios, simulations, screen captures, animated gifs, checklists, e-books, articles, blog posts, interviews etc. as study material or lectures, assignments, projects, test questions, question-answer bank, practice exercises. We focus on self-paced learning where the involvement of instructor-led learning is completely absent. However, the learner-content interaction has not been keenly focused on and widely explored in the past by the researchers. E-learning content may further be categorized into two primary elements, learning content and website content. “Learning content” denotes comprehensive and accurate study material delivered to the learners in a concise and well-timed fashion. The content is majorly distributed through the electronic channels by employing the internet, satellite TV, radio, and storage devices like compact discs, hard-discs, and pen-drives, etc., (Bates, 2005). The effectiveness of this is predominantly bolstered upon the overall quality of the electronic-based learning systems through various means including digital collaborations and virtual classrooms.

Website content, one major variable of the e-learning service quality (Udo, Bagchi, & Kirs, 2011), signifies the learning content accessible to the learners via the website platform, at any time and in an accurate and concise form. As visualization has become of utmost importance in this present day technological learning environment (Udo & Marquis, 2002), so now the impetus of any service provider is on the website’s visual design, colours, layouts, fonts, and shapes, along with navigation aids such as taskbars, hyperlinks, and checkboxes (Robins & Holmes, 2008).

Satisfaction may be termed as the customer’s fulfilment response to a service obtained from its evaluation or emotional assessment. It is a representation of the belief about the service that it is leading to a positive feeling. Learners’ satisfaction can be defined as a short-term mindset resulting from self-assessment of their educational experiences, services, and facilities (Weerasinghe & Fernando, 2017). The key factors of enhancing e-learning are Multimedia (Liaw, 2008; Liaw & Huang, 2013), learning content and website content (Uppal, Ali, & Gulliver, 2018), interaction (Bolliger, 2004) along with the variables of SERVQUAL model viz., assurance, responsiveness, tangibility (Saxena, Baber, & Kumar, 2020; Udo et al., 2011; Uppal et al., 2018). Also, to ensure a successful e-learning student experience, the learning content should be well designed, offering compatible technology with a range of learning management systems (Gudanescu, 2010; Koller, Harvey, & Magnotta, 2008). The primary focus of such efforts is to develop a sense of engagement among the learners by interacting with the course content in a meaningful way to understand the course structure.

The advancements in information technology and continuous involvement of the students in the online learning system continues to develop new avenues and openings for mutual interactions, which calls for the purposeful selection of activities fostering learner satisfaction. As the options for interaction through the content within the e-learning environment grow, so does the necessity to assess the impact of content on E-learning satisfaction. This rationale incites the researchers to examine this aspect of e-learning quality and the following hypothesis is developed:

- *H1: E-learning Content* (*eLC*) *has a significant effect on student satisfaction* (*SAT*)

### E-learning content (*eLC*) and e-learning quality (*eLQ*) of the learners

E-learning quality is a complex and multidimensional topic, as it is difficult to gauge it’s all its aspects to assure learning excellence. There are certain unique components of e-learning that are crucial to assess its performance, (Jung, 2011). Phipps and Merisotis (2000) have identified the important factors in defining the high quality of online education including infrastructural support, course creation, teaching/learning pedagogy, learner and instructor assistance, and quality appraisal and assessment. Material/Content is also one of the ten quality assessment dimensions of the e-learning quality model as proposed by The Swedish National Agency for Higher Education (2008). Gillis (2000) also evaluated the quality of e-learning using content quality and usability as one of its dimensions of assessment models. Ehlers (2004) also ascertained the learning content as one of the important factors in assessing the quality of e-learning. Sun, Tsai, Finger, Chen, and Yeh (2008) identified the website content as an important dimension in e-learning service quality, along with accuracy, visualization, and aesthetics of the content. Reisetter, LaPointe, and Korcuska (2007) found that course content was the most important factor determining the quality of e-learning. An effective website content leads to a positive attitude of the viewer’s resulting in satisfaction with the web-based services, (Koernig, 2003). This poses for a study on the effect of the E-learning Content and E-learning Quality of the learners, and the formulated hypothesis is:

- *H2: E-learning Content* (*eLC*) *has a significant effect on E-learning quality* (*eLQ*)

### E-learning quality (*eLQ*) and e-learning satisfaction (*SAT*) of the learners

E-learning Quality may be defined as the design of the e-Learning experience, the contextualized experience of learners, and evidence of learning outcomes (Jung, 2011). Dondi, Moretti, and Nascimbeni (2006) identify students as the key players in assessing the quality of e-learning and integrate learners’ responses in the framework they call Sustainable Environment for the Evaluation of Quality in E-Learning (SEEQUEL), to assess learner satisfaction. Several studies in the past have validated that e-learning service quality affects e-learning student satisfaction (Al-Rahmi et al., 2018; Pham, Limbu, Bui, Nguyen, & Pham, 2019). Further, in the e-learning environment, universities are required to continually improve the quality of e-learning services to bring satisfaction to the learners (Lee, 2010). Baber (2020) found students perceived learning as a determinant of student satisfaction during the COVID-19 pandemic. Consequently, the effect of e-learning Quality on the satisfaction of e-learning is assessed by setting the hypothesis:

- *H3: E-learning quality has a significant effect on E-learning satisfaction*

### COVID-19 perceived harm (*CoPH*) and *eLQ-SAT*

The COVID-19 pandemic has considerably impacted the thinking and consumption patterns of the present world people. Educational institutions are facing lockdowns to curb the further spread of the Coronavirus. The shutdown of educational institutes aiming at the public health emergency is a contagion effect of the coronavirus. In response, the academic institutions and learners are resorting to e-learning so that learning processes are not largely affected. Perceived harm of any type is interconnected with the emotional and psychological behavior of the people. Individual perceptions and cognitions are positively associated with the perceived severity and expected impact of this harm. The perceived harm of the virus is expected to influence the emotions & judgments of the learners (Constantin & Cuadrado, 2019; Murray & Schaller, 2016). Chen et al. (2020) examined the different factors of online education platforms during the adverse period of the virus and found its positive effect on learner satisfaction. The online learning platform availability is found to be the most important factor resulting in the satisfaction of students. Zitek and Schlund (2021) suggested that the perceived impact of COVID-19 on daily life is associated with greater health anxiety. Baber (2021) found the moderating effect of perception about maintaining social distance reduces the effect of social interaction on the effectiveness of online learning during the COVID-19. Saxena et al. (2020) studied the quality of e-learning and its impact on the satisfaction of e-learners under the moderating influence of perceived harm of the COVID-19 virus and found it mostly insignificant. It is expected that the perceived harm of the disease will positively influence the e-learning perception of the people. The moderating role of the perceived harm in the e-learning quality and e-learning satisfaction is yet to be explored, so the following hypothesis is devised in this direction:

- *H4: Perceived harm of getting COVID-19 significantly moderates the relationship between Content and E-learning quality*

### Interrelationship between *eLQ*, *eLC-SAT*

Saxena et al. (2020) found e-learning Content has no significance on the e-learning quality during the COVID-19 pandemic. The e-learning quality is also expected to play the role of a mediator for the relationship between E-learning Content and E-learning Quality, therefore the hypothesis designed to assess this aspect is:

- *H5: E-learning quality significantly mediates the relationship between Content and student satisfaction*

## Method

### Data collection and instrument

**Fig. 1:**
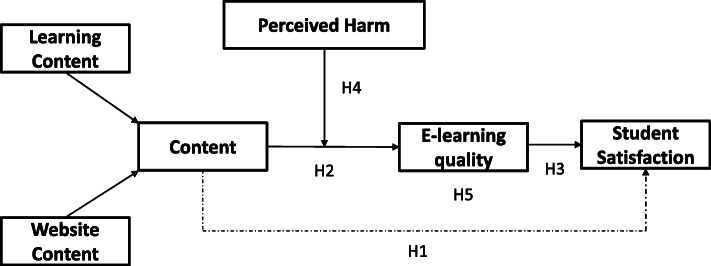
Research Model

The data is collected through a structured questionnaire on a 5-point Likert scale wherein responses of 435 undergraduate and graduate management students (International and national) in Indian Universities. Due to the current COVID-19 situation of restricted physical movement, the questionnaires were administrated online through e-mails and Google-form links. An online version of the questionnaire was sent to the under-graduate and postgraduate students, accompanied by a cover letter. A snowball sampling approach was used to gather data within our network, which was further shared in the other networks. A conceptual model framework is proposed for understanding the relationship between the content of learning in an online environment and student satisfaction and the mediating effect of the e-learning quality. The perceived harm of being on campus is used as a moderating variable between e-learning quality and student satisfaction. The research model of the study is shown in Fig. 1. The questionnaire was written in English and the constructs used are shown in Fig. 1. The items of the constructs are taken from previous studies- learning content (Cheng, 2012), website content (Uppal et al., 2018), e-learning quality (Chen et al., 2020; Shahzad, Hassan, Aremu, Hussain, & Lodhi, 2020), student satisfaction (Baber, 2020) and perceived harm (Kleczkowski, Maharaj, Rasmussen, Williams, & Cairns, 2015) as shown in table 8. The content variable was formulated as a second-order construct from the first-order constructs of learning content and website content. The study uses the Partial Least Square (PLS) structural equation modeling (SEM) approach, a non-parametric method based on total variance, using the SmartPLS software 3.2.

### Demographic profile of learners

Based on the demographic information in Table 1, the maximum number of learners (72.4%) lies in the range of 22–25 years of age. Among the total respondents, 48.7% of students are male and the rest of the 51.3 students are females. 51.5% of students are Indians whereas 48.5% are international students of the university whose responses are recorded. Around 73% of students have enough experience in online learning.

**Table 1:** Demographic profile of learners

Category	Frequency	Percentage
**Age**		
18–22	90	20.7
22–26	315	72.4
26–30	25	5.7
Over 30	5	1.1
**Gender**		
Male	212	48.7
Female	123	51.3
**Nationality**		
Indian	224	51.49
Afghanistan	43	9.89
Africa	34	7.82
Bangladesh	28	6.44
Bhutan	36	8.28
Namibia	18	4.14
Nepal	39	8.97
South Korea	13	2.99
**Level of Education**		
UG	90	20.7
PG	345	79.3
**Experience in Online Learning**		
None	20	4.6
Not Much	98	22.5
Enough	317	72.9

### Measurement model assessment

For complex structures and data lacking normality, PLS-SEM is useful to test a research framework (Hair, Risher, Sarstedt, & Ringle, 2019). The factor loadings, alpha value, composite reliability, and average variance extracted (AVE) are shown in Table 2. All the values of factor loadings were meeting the minimum threshold value of 0.7 (Hair et al., 2019) except the second of item student satisfaction, which was deleted for further analysis. The third item of the student satisfaction variable’s factor loading was slightly less than 0.7, which was accepted. The values of reliability measurements-Cronbachs’ alpha and composite are also above the threshold level of 0.7 (Hair et al., 2019). To check the validity of data, Convergent validity measurement was checked through the Average variance extracted (AVE) and all the values are above the minimum level of 0.5 (Hair et al., 2019).

**Table 2:** Measurement model

Measurement items^a^	Factor Loadings	Cronbach Alpha	Composite Reliability	AVE
**Learning Content**		**0.856**	**0.913**	**0.779**
Sufficient Learning Material	**0.807**			
Updated Information.	**0.906**			
Needful Content	**0.930**			
**Web site content**		**0.945**	**0.958**	**0.820**
Audio And Video Elements	**0.881**			
Animations/Graphics And Multimedia	**0.904**			
Relevant Course Information	**0.924**			
Easily Accessed And Navigated Website	**0.922**			
High-Quality Information	**0.897**			
**E-Learning Quality**		**0.828**	**0.885**	**0.660**
Overall Quality of the Instruction	**0.776**			
Overall E-Service Quality	**0.858**			
Overall System Quality	**0.859**			
Overall Information Quality	**0.750**			
**Student Satisfaction**		**0.803**	**0.878**	**0.710**
Decision Making	**0.909**			
Choice to Enroll	**Deleted**			
Spent on Right Thing	**0.658**			
Enjoyable Learning	**0.933**			
**Perceived Harm**		**0.727**	**0.846**	**0.646**
Lessen the chance of disease development	**0.755**			
Requisite in the current period	**0.807**			
Confidently Engaged in Social Distancing	**0.847**			

^a^Detailed items are shown in Table 8 in Appendix

### Divergent validity

It is important to measure the divergent validity of each construct to be sure that constructs that were supposed to be different from each other are actually different. Fornell-Larcker criteria for divergent validity were established as the correlations between the constructs are lower than the square of the AVE’s as shown in Table 3. (Fornell & Larcker, 1981). The HTMT ratio further endorses the divergent validity as all values shown in Table 4. are below the acceptable of value 0.85 (Henseler, Ringle, & Sarstedt, 2015).

**Table 3:** Fornell-Larcker criteria for divergent validity

	(LC)	(WC)	(ELQ)	(STS)	(PH)
**Learning Content (LC)**	**0.883**				
**Website content (WC)**	0.287	**0.906**			
**E-Learning Quality (ELQ)**	0.264	0.185	**0.812**		
**Student Satisfaction (STS)**	0.076	0.109	0.411	**0.842**	
**Perceived Harm (PH)**	0.177	0.156	0.498	0.267	**0.804**

**Table 4:** HTMT ratio for divergent validity

	(LC)	(WC)	(ELQ)	(STS)	(PH)
**Learning Content (LC)**					
**Website content (WC)**	**0.319**				
**E-Learning Quality (ELQ)**	**0.309**	**0.208**			
**Student Satisfaction (STS)**	**0.097**	**0.125**	**0.458**		
**Perceived Harm (PH)**	**0.229**	**0.188**	**0.64**	**0.331**	

## Data analysis and results

### Path coefficient results

The results signify the positive relationship between the content and e-learning quality (β: 0.254, *p* < 0.000) and e-learning quality and student satisfaction (β: 0.392, *p* < 0.000) as shown in Table 5. This implies that both the learning content and website content have a positive influence on e-learning quality and student satisfaction. The values of R^2^ on e-learning quality and student satisfaction are 0.064 and 0.178 which means 17% of the variance in student satisfaction is explained by the e-learning quality; supporting the results of previous studies (Koernig, 2003; Rhode, 2009; Udo et al., 2011). The implication of this result is that the instructors and administrators should pay attention to content development and design to enhance the quality of the e-learning system and student satisfaction.

**Table 5:** Estimated path relationships

Hypothesis	Path relationships	Original Sample (O)	T Values	***P*** Values	R square	Remark
**H1**	**Content**➔ **Student Satisfaction**	0.008	0.197	**0.844**	–	Not supported
**H2**	**Content**➔**E-learning quality**	0.254	5.730	**0.000**	0.064	Supported
**H3**	**E-learning quality**➔ **Student Satisfaction**	0.392	6.459	**0.000**	0.178	Supported

### Moderating role of perceive harm

The perceived harm has an insignificant moderating effect on student satisfaction as shown in Table 6. The value of significance is 0.410 and is more than 0.05 therefore we reject hypothesis 4. This means getting COVID-19 does not significantly moderates between content and e-learning quality and could be attributed to coincidental. The quality of e-learning has a positive significant relationship with learners’ satisfaction and this relationship is not affected by the threat of being infected on the campus during the pandemic of COVID-19. It was expected that threat of catching the virus on campus will make students satisfied with whatever e-learning quality they are getting during the sudden shift towards e-learning. However, this threat is not affecting the relationship between quality and student satisfaction. To make students satisfy, the highest level of quality in e-learning has to be maintained.

**Table 6:** Moderation effect

Hypothesis	Path relationships	Original Sample (O)	T Values	***P*** Values	Remark
H4	Perceived Harm * E-learning quality ➔Student Satisfaction	0.052	0.824	0.410	Not supported

### Mediating role of e-learning quality

**Fig. 2:**
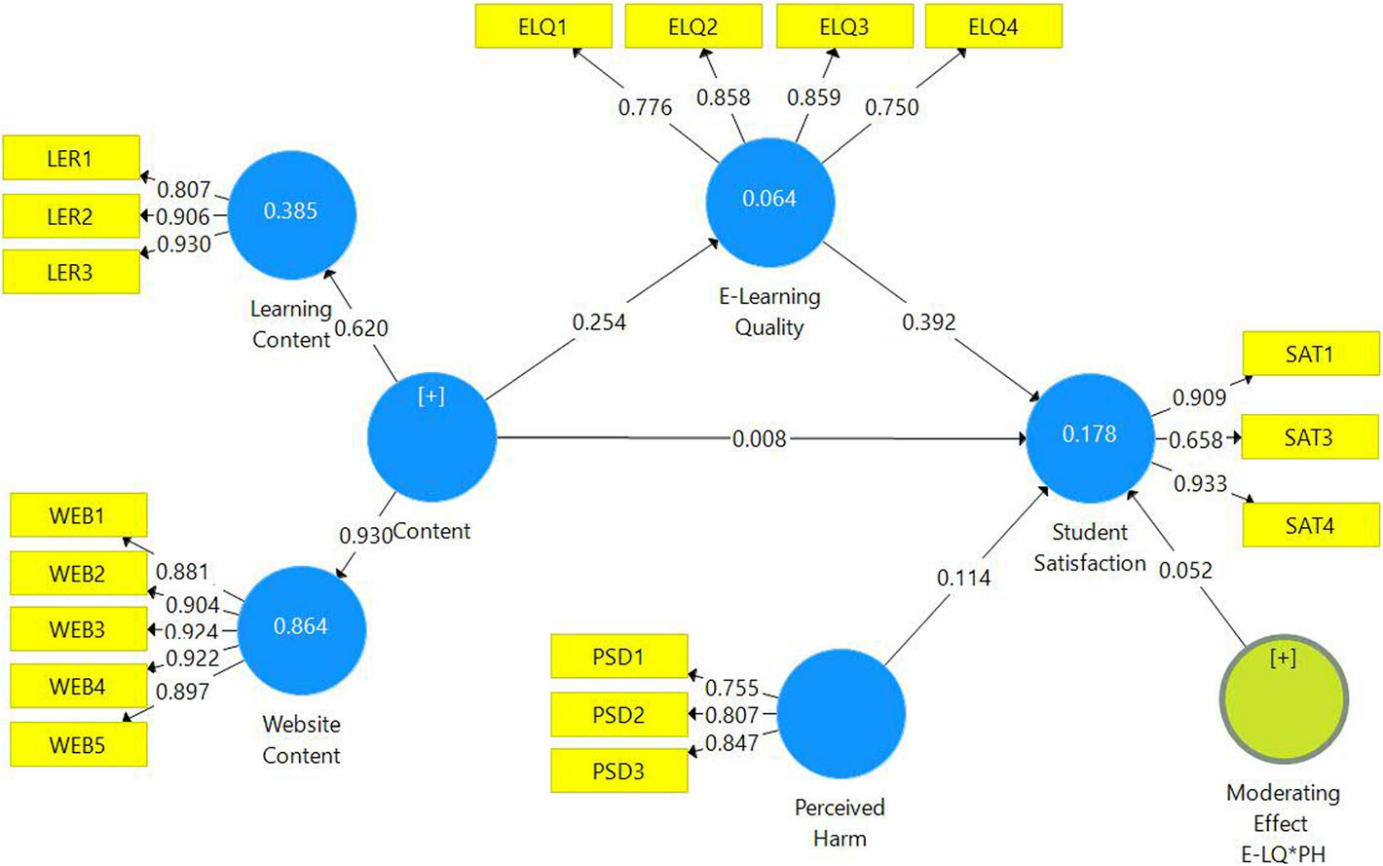
Path Coefficients

The mediating role of e-learning quality is significant between content and student satisfaction (β: 0.099, *p* < 0.000) (Table 7). The value of significance is 0.000 and is less than 0.05 therefore we accept hypothesis 5. This means e-learning quality is imperative and the relationship between content and student satisfaction is very effective and a correlation exists. However, there is an insignificant direct relationship between content and student satisfaction. This means that the content is directly irrelevant to student satisfaction and only has a positive effect through the mediating role of e-quality learning. Therefore the results suggest the acceptance of hypotheses H2, H3 and H5 while rejecting H4 and H1. Figure 2 represents the estimated PLS-SEM model with path coefficients and *p* values.

**Table 7:** Mediation effect

Hypothesis	Path relationships	Estimates	T Values	***P*** Values	Remark
**H5**	Content ➔ E-learning quality➔ Student Satisfaction	0.099	3.969	**0.000**	Supported

## Discussion and implications

The COVID-19 outbreak has disrupted the education sector and enforce the students to engage in learning via an online study platform. The major academic institutions of the world, during this pandemic, are now offering students the option of getting an education by signing up for online courses and earning the necessary credits. Continuing the education during the pandemic through e-learning, has become equally important for the instructors to enable the interaction of students with the course content in a more meaningful way. The content and course material for e-learning should be designed in such a way that it could enhance the perception of system usefulness (Lee et al., 2009).

The current study was focused on assessing the mediating role of e-learning quality between e-content and learner satisfaction and has given noteworthy implications in the field of online education. The results of the study depict that both the learning content and website content provided under the online study environment are important factors of e-learning quality, having a positive effect on the e-learning quality and student satisfaction. Thus, the instructors and administrators should pay attention to the content development and designing of the course structure in order to develop a sense of engagement among the learners to understand the course structure and, to ensure the quality of the e-learning system and student satisfaction. The website content provided in the e-learning platform should be easy to navigate and provide useful information as it is the only interface between the learner and instructor during the current times of a pandemic (Saxena et al., 2020). Besides, the relationship between the quality of e-learning and Learners’ satisfaction is not affected by the threat of being infected on the campus during the pandemic of COVID-19. Thus, to make students satisfy, it is important to maintain the highest level of quality in e-learning. It was expected that threat of catching the virus in offline classes will make students satisfied with whatever e-learning quality they are getting during the sudden shift towards e-learning. The quality of e-learning has a positive significant relationship with Learners’ satisfaction and this relationship is not affected by the threat of being infected on the campus during the pandemic of COVID-19. Grabbing and holding the attention of students needs quality in e-learning along with the effective website and learning content containing infographics, video clips, forums, and quizzes to enable them to think out of the box. The instructors and web designers should incorporate these tools to make an effective online learning program along with providing quality in e-learning.

It is significant for the content designers and the instructors to know the perceived level of satisfaction of the learners with the current content and e-learning quality provided by the various online courses. Effective website content can lead to a positive attitude of the viewers and resulting in the satisfaction of learners. In addition to that, e-learning quality plays a mediating role between content and student satisfaction along with content as directly irrelevant to student satisfaction proves that not only content creation is important, but to enhance student satisfaction the instructor should focus on the quality of e-learning. Thus, e-learning strongly influences learner satisfaction as a mediator between the content and learner satisfaction.

## Limitations and future directions

Although the present study has a significant contribution to understanding the role of e-content and e-learning quality in satisfying the students, however, this study also has few limitations which could be minimized in further studies. The current study has made all efforts to solicit the responses of university students belonging to different countries. The maximum number of students in the survey had prior online learning experience; a future study can be conducted by including only those students who have shifted from the traditional classroom classes to the virtual classes. On one side e-learning is playing a significant role in the education stream, especially in this era of the pandemic, on the other side there are several issues and challenges in front of the universities like the availability of the internet, sufficient learning resources. Moreover, future studies can be conducted from the perspective of teachers and instructors, to understand the pitfalls of the online education system.

## Conclusion

COVID-19 pandemic has placed an urgent worldwide need to adopt the new technology learning system, in order to continue the learning process. E-learning quality is imperative and plays a significant mediating role in the Content and Learners’ satisfaction. The quality of e-learning has a positive influence on the satisfaction of students, but this relationship is not affected by the threat of a pandemic. E-learning content and e-learning quality have emerged as a solution to minimize the disruption of education and satisfy the students. The threat of catching the virus on campus has compelled the students to depend on the online learning environment, but such a threat does not imply satisfaction of learners with online education, although it is the e-learning quality that plays a major contribution in the learners’ satisfaction. Thus, the findings of the study suggest that in order to make students satisfy, the highest level of quality in e-learning has to be maintained by the instructors, administrators as well as institutions.

## Data Availability

Not applicable.

## Appendix

**Table 8:** Instrument items

**Learning Content**	
The e-learning system provides me with sufficient learning content.	
The e-learning system often provides updated information.	
The e-learning system provides the learning content that I need.	
**Web site content**	
The web site uses audio and video elements properly.	
The web site uses animations/graphics and multimedia features properly.	
The course website has relevant course information and learning material.	
The course website can be easily accessed and navigated	
The website provides high-quality information	
**E-Learning Quality**	
The overall quality of the instruction I get from online learning is (poor-excellent)	
The instructional web site seems to be up to date	
The instructional web site works well	
The instructional web site has clear instruction	
**Student Satisfaction**	
Would you agree to say that “I am satisfied with my decision to enroll in the online classes?”	
Would you agree to say that “My choice to enroll in online classes was a wise one?”	
Would you agree to say that “I think I did the right thing when I paid for online learning service?”	
Would you agree to say that “I feel that my experience with online learning has been enjoyable?”	
**Perceived Harm**	
If I were to engage in social distancing (e.g. by avoiding public transport and social events) I would lessen my chance of developing an infectious disease.	
I am encouraged by engaging in social distancing during times of infectious disease because I feel it would be a necessity to do it.	
I feel confident in my ability to engage in social distancing during times of infectious disease.	

## Abbreviations

HTMT: Heterotrait-monotrait
PLS-SEM: Partial least squares structural equation modeling
